# An in silico protocol for predicting genetic biomarkers in rare diseases: a case study in sporadic amyotrophic lateral sclerosis

**DOI:** 10.3389/fgene.2026.1742595

**Published:** 2026-03-12

**Authors:** Ali Aguerd, Badreddine Nouadi, Abdelkarim Ezaouine, Imad Fenjar, Faiza Bennis, Fatima Chegdani

**Affiliations:** Laboratory of Integrative Biology, Faculty of Science Ain Chock, University Hassan II, Casablanca, Morocco

**Keywords:** genetic biomarkers, genome-wide-associations studies (GWAS), *in silico* prediction, machine learning, rare diseases, single nucleotide polymorphisms (SNPs), sporadic amyotrophic lateral sclerosis (SALS)

## Abstract

Studying the genetics of rare diseases is challenging because small sample sizes limit the statistical power of standard methods like Genome-wide association studies (GWAS). We created a new machine-learning approach to find candidate Single Nucleotide Polymorphisms (SNPs) when data is scarce. Our method trains a Random Forest model to spot similarities between SNPs. We used 189 known Sporadic Amyotrophic Lateral Sclerosis (sALS)-linked SNPs as positive examples and 938,544 unrelated SNPs as negatives. The model learns from genomic location, significance levels, nearby genes, and other features. When we tested it on sALS, it performed exceptionally well, with 93.8% accuracy and near-perfect AUC scores. The method uncovered 1,890 new SNP candidates for sALS. Among these, 209 reached genome-wide significance, and 50 appeared repeatedly in our analyses, making them strong candidates. Key genes like *SARM1*, *OPHN1*, and *BPTF* emerged from the results, all connected to neural health and survival pathways. Our examination revealed a notable excess of SNPs on chromosome 18 compared to expectations. This non-random distribution underscores the region’s particular interest. Here, our approach demonstrates its ability to extract meaningful signals from a restricted sample. The results generated by this approach enable early diagnosis of the disease under study, explanation of its mechanism, and identification of therapeutic targets.

## 1 Introduction

Rare diseases are defined by low prevalence, for example, affecting fewer than 200,000 people in the United States, less than 5 per 10,000 people in Europe, and fewer than 50,000 individuals in Japan. There are currently around 9,600 recognized rare diseases, with a cumulative prevalence estimated between 1.5 and 6.2 percent of the population. Mortality is high in certain groups, and these diseases also impose substantial economic burdens, with lifetime costs ranging from 133,000 to nearly 2 million euros (Ferreira, 2019). Among these conditions, Sporadic Amyotrophic Lateral Sclerosis (sALS), also known as Lou Gehrig’s disease, is a progressive neurodegenerative disorder. It primarily affects the motor system. Over time, patients experience a gradual loss of muscle control. This includes difficulties with speech and swallowing (Saumitra et al., 2025). Its global incidence is estimated at approximately 1.68 cases per 100,000 person-years (Pang et al., 2025). Like all rare diseases, sALS suffers from a lack of genetic data due to its low incidence. Indeed, with very limited genetic data, it is impossible to fully understand, diagnose early, and treat this rare disease effectively.

Single Nucleotide Polymorphisms (SNPs) play an important role in studying rare diseases (Fadason et al., 2022). They can act as biomarkers that help reveal genetic susceptibility and also provide insights into molecular pathways involved. Unfortunately, the rarity of some diseases such as sALS has limited the number of their characteristic SNPs.

Several methodological approaches have linked SNPs to disease, especially genome-wide association studies (GWAS) (Ren et al., 2023) and Fine-mapping (Schaid et al., 2018). Yet, they often fail to identify causal SNPs in rare cases, due to small samples and high linkage disequilibrium in some genetic regions (Schaid et al., 2018). As a result, in the case of sALS, diagnosis is often delayed. Current therapies only slow disease progression and relieve symptoms. No molecular pathway fully explains its pathogenesis (Saumitra et al., 2025; Pang et al., 2025).

The above information shows the importance of a technique that combines independence from large amounts of data and precision. Machine learning aligns perfectly with these two requirements. In fact, it learns the link between disease and its genetic data, even if limited, in order to predict new genetic associations. This helps uncover the missing heritability of rare disease (Jin et al., 2024).

The present work outlines an ML-based predictive protocol designed to identify characteristic biomarkers. This approach is based on the assumption of genetic proximity: two SNPs located in close genomic regions are often in linkage disequilibrium and tend to exert similar effects. Therefore, they may be associated with the same chronic pathology (Slatkin, 2008; Sved and Hill, 2018). This biological rationale underpins the integration of genomic position indicators as key predictive features. This study increases the pool of diseases-related SNPs and creates new opportunities to improve diagnosis. It helps construct relevant signaling pathways and also facilitates the search for targeted therapeutic molecules.

This *in silico* protocol provides a scalable framework that can uncover new genetic associations and improve our understanding of rare diseases. Unlike standard applications of machine learning in genetics, which directly classify individual SNPs—a strategy requiring a large number of positive examples—our protocol recasting the task as a pairwise similarity prediction problem. His approach uses the limited known SNPs to train the model to identify genomic proximity patterns, based on the biological premise that neighboring SNPs often have similar functional effects.

## 2 Materials and equipment

### 2.1 Computational environment

We used Python 3.12.6 for all analyses and PyCharm as the development environment. Python was chosen because it offers many scientific libraries adapted to our context. The computations were done on a desktop with an Intel Core i5 processor and 16 GB of RAM. This setup handled all machine learning tasks on the genomic dataset without performance limitations.

### 2.2 Core python libraries for data management and analysis

We used the pandas library (Sved and Hill, 2018; McKinney, 2010; Pandas documentation, 2009) as the main tool for handling and preparing our data. It allowed us to combine different genomic datasets, fill in missing values, create new features from genomic metrics, organize genetic annotations, and filter variants efficiently. At the same time, numpy (Harris et al., 2020) supported all numerical calculations. It made array operations on genetic matrices easy, enabled mathematical transformations and linear algebra needed for the analyses, and provided efficient structures for handling the SNP matrices and feature sets in this study.

### 2.3 Machine learning framework

We used scikit-learn (Harris et al., 2020; Category Encoders, 2013; Pedregosa et al., 2011) as our main machine learning tool, specifically employing Random Forest Classifier for SNP similarity prediction and model evaluation.

### 2.4 Visualization and results interpretation

We used matplotlib (Hunter, 2007) and seaborn (Waskom, 2021) to visualize our results. They allowed us to plot the main performance metrics, including the ROC curve, the Precision–Recall curve, and the confusion matrix. These plots helped us see how the classifier performed and judge the reliability of its predictions. Additional figures generated from the predicted SNPs were also produced to explore their genomic distribution and biological relevance.

### 2.5 Supplementary libraries

Additional Python libraries helped with other tasks. We used joblib to save and reload models. tqdm showed progress bars during iterations. The built-in warnings module managed any warnings during execution.

## 3 Methods

### 3.1 Data collection

Positive cases comprised 189 sALS-associated SNPs obtained from the GWAS Catalog (Cerezo et al., 2025) in TSV format. Negative cases consisted of 938,544 SNPs, also retrieved from the GWAS Catalog, which were initially associated with other traits (non-sALS) and selected to represent well-characterized, biologically active loci. For each SNP, genomic features including mapped gene(s), chromosomal location, and associated p-value were extracted.

The GWAS Catalog was selected because it provides curated, standardized, and biologically meaningful SNP annotations, ensuring that both positive and negative sets are well-characterized and compatible with the analysis pipeline. The protocol is fully reproducible with SNP datasets from any source, provided that the input files include the required columns for the Python pipeline. Specifically:

- Positive TSV file must contain the following columns: riskAllele, locations (chromosome:position), mappedGenes, and pValue.
- Negative/candidate TSV file must contain the following columns: SNPs (or STRONGEST SNP-RISK ALLELE), CHR_ID, CHR_POS, MAPPED_GENE, P-VALUE, and DISEASE/TRAIT.

### 3.2 Data preprocessing

For model training, example pairs were constructed: similar pairs (positive-positive SNP pairs) and dissimilar pairs (positive-negative SNP pairs). Five differential features were computed for each pair: chromosomal discrepancy, positional distance, transformed p-value difference (-log10), divergence in associated gene count, and intergenic status difference. These dissimilarity features served as input variables for the random forest model.

For SNPs on the same chromosome (chr_diff = 0), the positional distance (pos_diff) provides intra-chromosomal localization, contributing to the assessment of similarity alongside other features. For SNPs on different chromosomes (chr_diff ≠ 0), the chromosomal discrepancy itself indicates positional dissimilarity; the model then further evaluates similarity based on the other features.

Before training the model, the SNP data were preprocessed to ensure consistency and machine-readability. Missing or non-numeric values for positions and p-values were corrected. Chromosomes were converted to numerical codes. Simple gene-related features were also computed, such as the number of mapped genes and whether the SNP was intergenic.

### 3.3 Machine learning model building

We built a Random Forest model (Breiman, 2001), whose characteristics are summarized in Table 1, to predict novel SNPs potentially associated with sALS. The model identifies, among non-associated SNPs those most similar to known sALS-associated SNPs. Leveraging the assumption of genetic proximity. Our methodological approach does not use the classic Random Forest approach. Instead of simply classifying SNPs, the proposed protocol performs a pairwise comparison (positive SNP–candidate SNP) based on the genomic context. This methodological principle transfers the classification problem to a pairwise comparison, which allows for indirect classification (sALS, non-sALS). As a result, the reliability of predictions is no longer limited by sample size.

**TABLE 1:** Model characteristics.

Element	Description
Model type	Random forest classifier
Parameters	100 trees, max depth = 5
Class balancing	Yes
Data split	80% training/20% testing

To achieve its objective, the model relies on the basic features summarized in Table 2, while enhancing prediction reliability through feature engineering procedures detailed in Table 3.

**TABLE 2:** Features used in training.

Feature name	Source data	Description
rsID	riskAllele/SNPS	SNP identifier extracted from source data (e.g., rs12345)
Chromosome	Locations/CHR_ID	Chromosome number (e.g., X, 2, MT)
Position	Locations/CHR_POS	Genomic position on the chromosome (numeric)
MappedGenes	mappedGenes	Gene(s) associated with the SNP (comma-separated if multiple)
pValue	P-VALUE	Statistical significance of the SNP–trait association
Target	*Derived*	Binary label: 1 for SNPs associated with sporadic ALS, 0 for SNPs not associated with sporadic ALS.

**TABLE 3:** Feature engineering summary.

Feature name	Engineering steps	Purpose
Per-SNP feature — gene_count	Count genes in mappedGenes (e.g., “GENE1,GENE2” → 2), 0 if no gene data	Quantify gene complexity near the SNP.
Per-SNP feature — is_intergenic	1 if mappedGenes is NaN, else 0	Identify intergenic regions
Per-SNP feature — log_pvalue	Convert pValue to numeric; replace 0 with 1e-300; compute -log10 (pValue)	Handle skewed p-value distribution and emphasize significance
Per-SNP feature — chr_encoded	Map chromosomes: 1–22 → 1-22, X→23, Y→24, MT→25, others→26	Encode chromosomes numerically for modeling
Pairwise feature — chr_diff	abs (ref_chr_encoded - cand_chr_encoded)	Measure chromosome proximity
Pairwise feature — pos_diff	abs (ref_position - cand_position)	Measure closeness in genomic position
Pairwise feature — pval_diff	abs (ref_log_pvalue - cand_log_pvalue)	Compare statistical significance
Pairwise feature — gene_diff	abs (ref_gene_count - cand_gene_count)	Compare gene association complexity
Pairwise feature — intergenic_diff	abs (ref_is_intergenic - cand_is_intergenic)	Check if both SNPs are intergenic (0) or not (1)

The Random Forest parameters were selected to achieve an optimal balance between predictive performance, generalization capacity, and computational efficiency, ensuring the protocol’s reproducibility for other rare diseases. One hundred trees (n_estimators = 100) were used, as this number provided sufficient variance reduction and prediction stability without unnecessary computational overhead. The maximum tree depth was limited to five (max_depth = 5), a conservative threshold that effectively prevents overfitting while still capturing the non-linear interactions between our five engineered features. Given the severe class imbalance inherent to rare disease genetics class weight balancing was activated to ensure the model adequately learned from the minority class. Finally, the dataset was split into 80% for training and 20% for testing to reduce the risk of overfitting. Model performance was evaluated using ROC–AUC, precision–recall curves, and classification reports. These results were further reinforced through more rigorous validation procedures, including cross-validation and external validation on independent rare disease datasets (Section 3.4). This parameter set was intentionally selected to be resource-efficient and fully reproducible, requiring only standard computational resources.

### 3.4 Model validation

Model validation is a crucial phase to ensure the reliable application of the generated predictions. While the hold-out validation method (80/20 split) serves as an essential initial filter against overfitting, complementary multi-level tests are indispensable for comprehensive validation.

To achieve this, we implemented a multi-step approach. First, a cross-validation of the primary task was conducted in two modalities: (1) on simulated data (500 SNPs generated with pre-defined similarity groups), where the Random Forest model, trained on 400 balanced pairs, demonstrated excellent ability to predict similarity on 200 test pairs; (2) through 5-fold cross-validation on real sALS/GWAS data, exactly replicating the main study protocol and confirming the model’s generalizability to unseen SNPs.

Second, a comparative analysis evaluated our Random Forest model against two supervised linear approaches (Ridge Regression and Logistic Regression) with equivalent regularization, using six complementary metrics (Accuracy, Precision, Recall, F1-Score, ROC-AUC, and Average Precision), confirming the superiority of the non-linear approach for this task.

Finally, external validation on distinct pathologies tested the model’s robustness on two additional rare diseases: Behçet’s syndrome (79 associations) and congenital heart malformation (301 associations). These pathologies, having respectively lower and higher numbers of associations than sALS (189 associations), allowed us to verify performance across a broad spectrum of data richness.

After confirming the model’s performance and reliability, it was then applied to predict novel SNPs. Specifically, the model generated 10 candidate SNPs for each of the 189 known sALS-associated SNPs, resulting in a total of 1,890 predicted SNPs.

### 3.5 SNP filtering and enrichment

After prediction, a TSV file was generated containing the predicted SNPs, along with their characteristics and those of the corresponding reference SNPs. Each row includes the reference and predicted rsIDs, their respective chromosome numbers and exact chromosomal positions, associated genes, p-values, and the computed similarity score. Although all predicted SNPs showed high similarity with known sALS-associated variants, we focused on the most significant and recurrent ones. Specifically, SNPs with a p-value corresponding to–log10(p) ≥ 7.3 (p ≤ 5 × 10^−8^) and/or predicted at least six times across all reference SNPs were retained. The first criterion corresponds to the standard genome-wide significance threshold used in GWAS studies, ensuring high statistical confidence (Fadista et al., 2016), (Liu et al., 2018; Sullivan et al., 2024). The second criterion was based on the observed distribution of prediction frequencies in our data: SNPs predicted 1–3 times were common and potentially noisy, 4–5 times were of intermediate frequency, whereas SNPs predicted six times or more were rare, statistically robust, and highly unlikely to occur by chance. We merged the predicted SNPs into final TSV files. Then, we added the reference and alternative alleles from Ensembl (Dyer et al., 2025). This approach ensured that retained SNPs were both statistically robust and biologically consistent, maximizing the likelihood of identifying variants with true functional relevance.

## 4 Results

### 4.1 Performance of the prediction model

**FIGURE 1:**
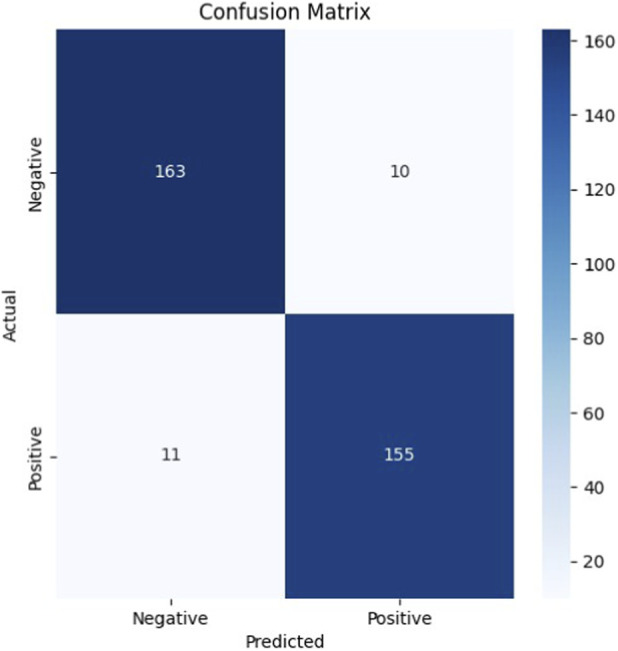
Confusion matrix showing the number of true and false classifications for both classes.

We evaluated the model using several metrics: accuracy, precision, recall, F1-score, ROC-AUC, and average precision. The confusion matrix in Figure 1 shows 163 true negatives, 155 true positives, 10 false positives, and 11 false negatives, giving an overall accuracy of 93.8%.

Table 4 provides the detailed classification report. Both classes show balanced performance. Precision was 0.94 for class 0 and 0.94 for class 1, while recall was 0.94 and 0.93, respectively. The F1-score, which balances precision and recall, was 0.94 for both classes. These results indicate that the model reliably distinguishes positive and negative cases.

**TABLE 4:** Classification performance report and key metrics.

Class	Precision	Recall	F1-score	Support
0	0.94	0.94	0.94	173
1	0.94	0.93	0.94	166
Accuracy	​	​	**0.94**	339
Macro avg	0.94	0.94	0.94	339
Weighted avg	0.94	0.94	0.94	339

Bold values indicate the overall accuracy of the model (rounded to 94 %).

**FIGURE 2:**
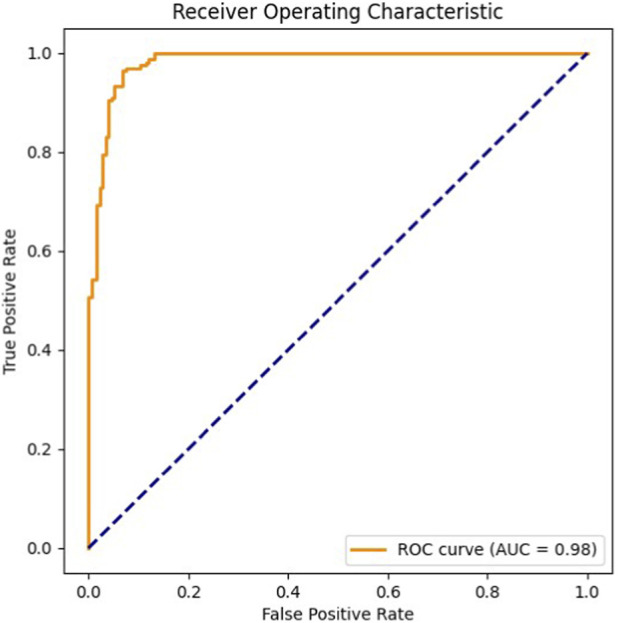
Receiver Operating Characteristic (ROC) curve showing the relationship between the true positive rate and the false positive rate (AUC = 0.98).

**FIGURE 3:**
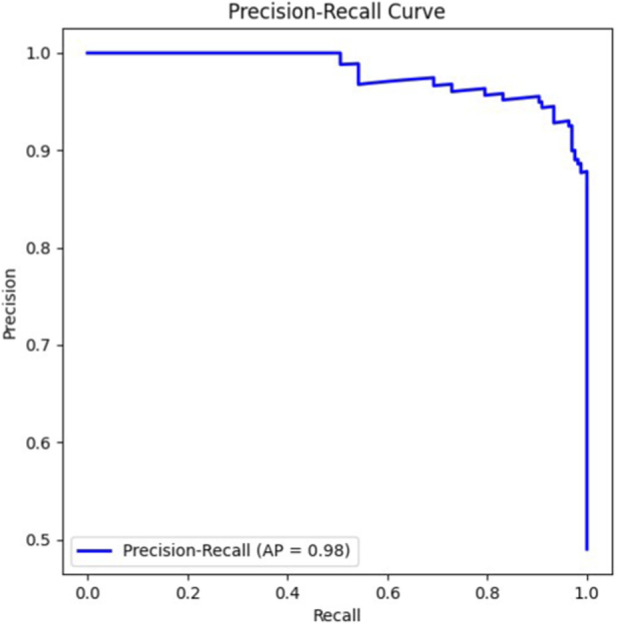
Precision–Recall (PR) curve showing the relationship between precision and recall (AP = 0.98).

The ROC curve in Figure 2 indicates an AUC of 0.983, reflecting strong class separation. Similarly, the Precision–Recall curve in Figure 3 shows an average precision of 0.979, demonstrating that the model maintains high precision even when recall varies.

The model’s performance was further validated through a series of rigorous tests. On simulated data, it achieved perfect discrimination (AUC = 1.000), confirming its foundational ability to learn similarity patterns. A five-fold cross-validation on the held-out sALS data yielded a mean ROC-AUC of 0.919 (±0.042), demonstrating robust generalization to unseen samples.

**FIGURE 4:**
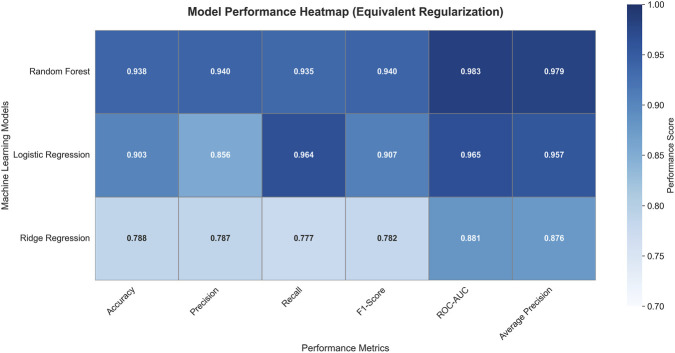
Performance comparison of machine learning models. The heatmap summarizes key evaluation metrics (Accuracy, Precision, Recall, F1-Score, ROC-AUC, and Average Precision) for the Random Forest, Logistic Regression, and Ridge Regression classifiers applied to the SNP similarity prediction task.

A comparative analysis against standard linear classifiers was conducted. The Random Forest model outperformed both Ridge Regression (ROC-AUC: 0.881) and Logistic Regression (ROC-AUC: 0.965), as detailed in Figure 4, justifying the selection of a non-linear, ensemble-based method for this task.

The model’s external validity was assessed by applying it to two independent rare diseases. It maintained strong predictive capability for Behçet’s syndrome (79 associations, ROC-AUC: 0.873) and congenital heart malformation (301 associations, ROC-AUC: 0.959), demonstrating consistent performance across datasets of varying sizes and etiologies.

In summary, the model demonstrates high predictive accuracy, robustness against overfitting, superiority to linear benchmarks, and reliable performance when transferred to other rare diseases, thereby validating its design and application for SNP similarity prediction.

### 4.2 Predicted SNPs

**FIGURE 5:**
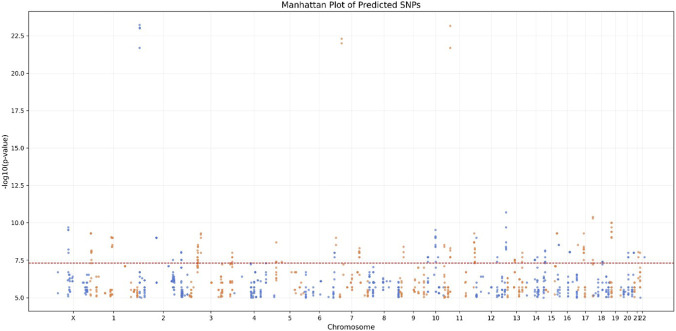
Manhattan plot of predicted SNPs. Each point represents a SNP positioned according to its chromosomal location (x-axis) and association strength (-log_10_ *P*-value) with sALS (y-axis). The horizontal red line indicates the genome-wide significance threshold (*P* < 5 × 10^−8^).

Our machine learning model enabled us to predict 1,890 SNPs with high similarity scores, ranging from 97.287% to 99.718%, relative to SNPs associated with sALS. Figure 5 shows their distribution according to their chromosomal position and corresponding -log10 (pValue).

**FIGURE 6:**
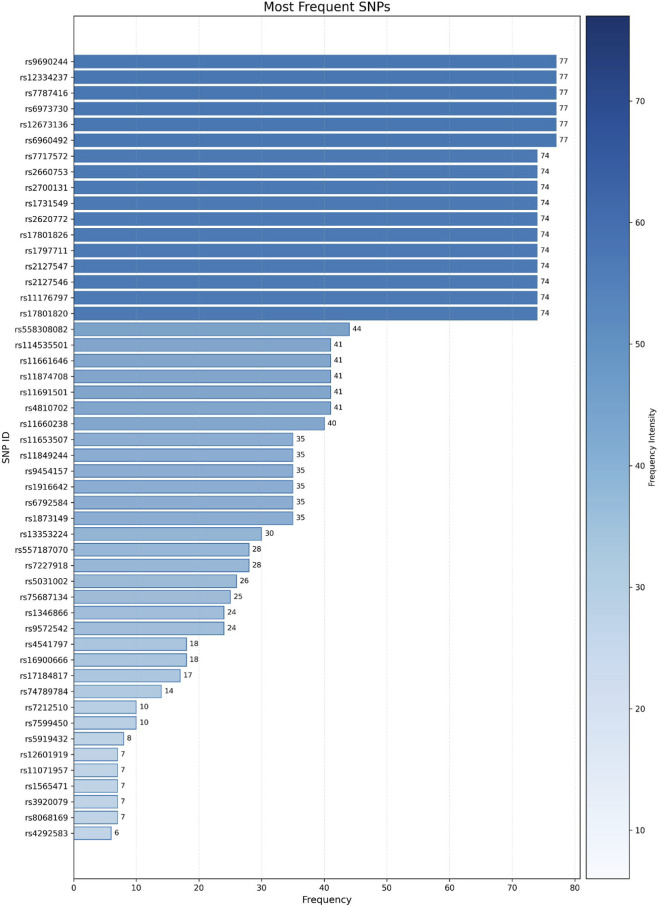
Frequency distribution of the 50 most recurrent SNPs predicted by machine learning for sporadic Amyotrophic Lateral Sclerosis (sALS). SNP identifiers are shown on the y-axis, with their corresponding prediction frequency intensity ranging from 6 to 77 on the x-axis.

Beyond statistical significance, several SNPs show a high occurrence frequency (Figure 6), which may reflect their potential involvement in sALS pathogenesis.

**FIGURE 7:**
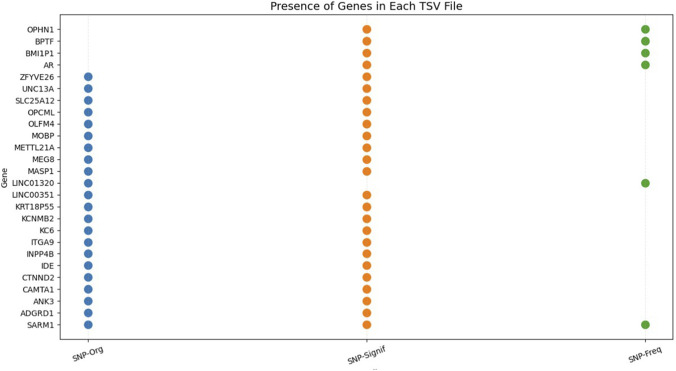
Characteristic Genes of sALS. Colored circles (•) denote gene presence in SNP categories: blue (SNP-Org, original confirmed *in vivo*), green (SNP-Freq, predicted frequent), orange (SNP-Signif, predicted significant).

We next analyzed the genes associated with the predicted SNPs, including both the significant and frequent variants, by categorizing them into three distinct groups (Figure 7).

**FIGURE 8:**
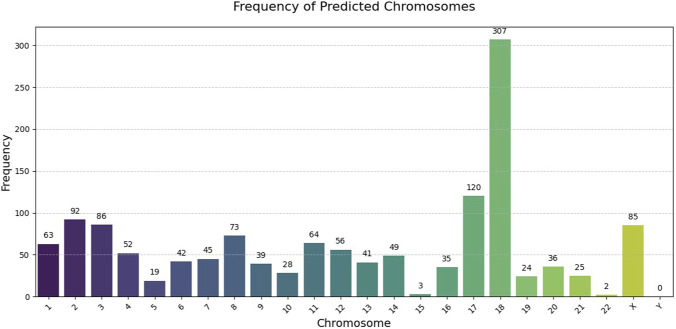
Genomic distribution of predicted sporadic ALS-associated SNPs across chromosomes. The histogram displays the frequency of predicted SNPs per chromosome, revealing chromosomes with the highest density of sALS-associated variants.

Finally, chromosome analysis of the predicted SNPs demonstrated the distribution frequencies illustrated in Figure 8.

### 4.3 Expected outcome of the protocol

In particular, this protocol will enable the expanding of sALS genetic database, which will deepen our understanding of this disease, as the predicted SNPs can be used to diagnose it. In addition, the interaction of genes carrying predicted SNPs may explain the molecular mechanism underlying this disease, which will facilitate the development of therapeutic strategies.

If we take this protocol in a general context, it can be used for any rare disease, generating genetic data that is useful for understanding, diagnosing, and treating them.

### 4.4 Advantages and limitations

Technically, the advantages of our protocol lie mainly in its simplicity, speed, and compatibility with all types of resources. Indeed, material resources hinder scientific research in several research laboratories, especially in developing countries. These key advantages are justified by the principle of prediction based on genetic approximation. This principle requires only position indicators for each SNP, which limits dependence on sophisticated resources. Biologically, the protocol used generates SNPs that can be interpreted at several levels (this point is detailed in Section 4.3).

Like all *in silico* studies, the results generated by this protocol require *in vitro* validation. This validation confirms the biological link with sALS or the disease studied by future applications. Furthermore, the application to sALS presented here relies on SNPs derived from diverse populations (e.g., European, Asian), which allows for the generalization of predictions regarding gene involvement in rare diseases and their extensions (gene interaction networks underlying the pathology and identification of potential therapeutic targets). However, this diversity can also limit the accuracy of population-specific diagnoses, as the same SNP may be benign in one population but pathogenic in another.

Our protocol does not include a dedicated linkage disequilibrium analysis; however, the candidate SNPs are lead variants from GWAS, representing the strongest statistical associations in their respective loci. While some may be in linkage disequilibrium with the true causal variants, their prior association with other traits confirms that they are not silent polymorphisms and tag potentially functional regions. Regardless of their linkage disequilibrium status, these SNPs provide information pointing to gene-level biomarkers, helping to guide the understanding of the rare disease under study. Nevertheless, predicted SNPs require further experimental validation, including linkage disequilibrium analysis, to confirm their biological relevance.

While increasing the number of positive SNPs may theoretically improve prediction accuracy, this is often constrained by the rarity of the disease and associated resource costs.

In summary, although our protocol offers a scalable method for biomarker discovery in rare diseases, these limitations highlight the importance of experimental validation and population-stratified analyses.

### 4.5 Possible pitfalls and artifacts

Our predictions are inherently dependent on the quality and completeness of the source GWAS annotations; any systematic biases, errors, or incomplete trait associations in the original data may propagate through our model and affect prediction reliability. High similarity to known SNPs does not always mean they are biologically relevant. Additionally, despite our multi-level validation and stringent filtering, there remains a possibility of false-positive predictions arising from random genomic correlations, linkage disequilibrium with true causal variants, or artifacts in repetitive genomic regions.

## 5 Discussion

### 5.1 Machine learning to overcome GWAS limitations in rare diseases

GWAS have contributed substantially to the identification of disease-associated polymorphisms, and fine-mapping has helped narrow these signals to variants that are more likely to be causal. Despite these strengths, both approaches show clear limitations when applied to rare conditions such as sALS. The statistical power they require depends on large cohorts, a requirement that is rarely met for low-prevalence disorders. In addition, variants with small effect sizes or those participating in multi-layered genetic interactions are often overlooked (Keller et al., 2014; Yousefian-Jazi et al., 2020). Together, these factors leave an important fraction of heritability unexplained and complicate the systematic discovery of genetic biomarkers in rare diseases.

To address these limitations, our approach introduces a methodological adaptation that applies machine learning specifically to the constraints of rare diseases. By transposing the biomarker discovery problem into a pairwise similarity task, and by designing differential features that explicitly encode genomic proximity, we circumvent the need for a large sample of associated SNPs. Machine learning offers a realistic alternative, particularly because it can capture non-linear relationships and polygenic effects even when the available data remain limited (Pasternack et al., 2025; Zhang et al., 2022; Grollemund et al., 2019). Indeed, working with limited data is already supported by several tools, such as the sequence kernel association test (SKAT) (Wu et al., 2011), as well as studies like (Wang et al., 2010; Enoma et al., 2022; Santorico and Hendricks, 2016).

Among the different algorithms that could be applied, we selected the Random Forest (RF) method. This choice was guided by its behavior with heterogeneous genomic features and its capacity to reduce overfitting through bootstrap aggregation and random sub-sampling of variables as well as by its superiority in this type of task compared with similar models, as already confirmed during the validation phase. In practice, RF constructs a collection of decision trees and aggregates their outputs, which tends to produce more reliable predictions when the underlying genetic signal is modest. Formally, the predicted class ŷ for a given sample x is obtained from the majority vote across the T trees composing the ensemble:

ŷ = mode{h_1_(x), h_2_(x), …, h_T(x)}

where *hᵢ(x)* represents the prediction of the *i*th tree. This ensemble behavior enables RF to capture epistatic interactions between SNPs—interactions that linear models tend to overlook. Several studies have shown that RF can exceed the performance of traditional statistical methods, both in terms of predictive accuracy and in the identification of relevant biomarkers (Wu et al., 2011; Wang et al., 2010).

In our case, the model reached strong predictive performance for sALS-associated SNPs, with an overall accuracy of 93.8% and well-balanced values for the main evaluation metrics (precision: 0.94, recall: 0.93, F1-score: 0.94). The discriminative ability of the classifier was also confirmed by the ROC curve (AUC = 0.983) and the Precision–Recall curve (AP = 0.979). These results likely reflect the effectiveness of the strategy adopted here: instead of treating the task as a classical classification problem, we framed SNP discovery as a similarity assessment, training the RF model to distinguish between pairs of SNPs based on a set of genomic features specifically engineered for this purpose.

When we examined the importance of different features, it became apparent that our approach captures meaningful biological information. Genomic distance between SNPs had the strongest influence on predictions, while differences in chromosome and in p-value came next. This pattern fits our hypothesis that SNPs close together are more likely to have related functions, likely due to linkage disequilibrium or shared regulatory elements. We deliberately calculated pairwise differences in position, chromosome, significance, and gene associations, so that the model would explicitly reflect these biological relationships. When tuning the Random Forest, we set the maximum tree depth to five and used 100 trees. This seemed to capture the key interactions between SNPs without overfitting to the training set. Because the dataset was highly imbalanced, we applied class weights to make sure that the rare sALS-associated variants were still recognized among the many negative cases. These settings were chosen to provide a reasonable trade-off between predictive performance and biological interpretability, while keeping the model behavior straightforward and transparent.

When looking at previous applications of RF in disease genetics, our model appears to perform very well. The AUC of 0.983 is higher than what has been reported for COPD (AUC = 0.766) (Yang et al., 2024) and Alzheimer’s disease (AUC = 0.92) (Jiao et al., 2025), although such comparisons should be made cautiously, given differences in datasets and methods. Even taking methodological differences into account, our results indicate that the similarity-based strategy works well. The way we constructed and combined features seems to have helped the model capture meaningful genetic patterns in sALS. Looking at the predictions, we noticed that some SNPs showed up repeatedly—in a few cases as many as 77 times. This repetition makes it more plausible that these variants are biologically meaningful and could be involved in the mechanisms underlying sALS.

Finally, despite the strong performance of our developed model, it is not intended to replace GWAS or fine-mapping. Instead, the proposed protocol serves as a complementary approach, building on the validated data these classical techniques provide. Its main strength lies in enriching and expanding the genetic information available for rare diseases by leveraging experimentally confirmed foundations.

This complementarity can be described in three key aspects. First, objectives differ: while GWAS aims to discover novel associations from population data and fine-mapping seeks to pinpoint causal variants within associated regions, our protocol focuses on expanding the candidate biomarker set from a limited number of known associations through similarity-based inference.

Second, the assumptions underlying the approaches are distinct. Our method relies on genetic proximity, assuming that nearby SNPs may have similar functional effects. In contrast, GWAS assumes common variants contribute to disease risk via detectable allele frequency differences, and fine-mapping assumes that causal variants are tagged by nearby SNPs through linkage disequilibrium.

Third, data requirements vary. Our approach operates effectively with a small set of known disease-associated SNPs and a background dataset, making it suitable for rare diseases. By comparison, GWAS requires large case-control cohorts for sufficient statistical power, and fine-mapping relies on high-resolution haplotype information and substantial sample sizes.

### 5.2 Reliability of predicted biomarkers

The 1,890 SNPs predicted by the RF model offer useful insights into the genetic landscape of sALS. Out of these, 209 variants reached conventional levels of statistical significance, while 50 were consistently identified across several prediction runs. This pattern suggests that these SNPs are more likely to be reproducible biomarkers rather than random hits. People who carry a larger number of these variants could be more prone to developing sALS. Such findings suggest that methods like machine learning can bring to light genetic factors that are otherwise difficult to detect in rare diseases.

Beyond their predictive accuracy, looking at the biological meaning of these SNPs highlights a number of genes that deserve particular attention. As illustrated in Figure 7, some genes appear across all three categories—the original SNPs confirmed experimentally, the predicted significant SNPs, and the frequently predicted SNPs—which supports the idea that they might play a causal role in sALS. It is worth noting that 22 of these genes have already been reported in previous studies as associated with sALS (Germain et al., 2025; Ma et al., 2022; Yang et al., 2024; Jiao et al., 2025; Benyamin et al., 2017), which adds further confidence that our model’s predictions reflect biologically relevant signals.

*SARM1* stands out among these genes because it shows up consistently across all SNP categories. This gene encodes a NAD^+^ hydrolase, which is essential for axonal degeneration after neuronal injury by regulating NAD^+^ metabolism (Sha et al., 2009; Gerdts et al., 2015; Fogh et al., 2016; Bloom et al., 2022; Giroud et al., 2025). Seeing it appear repeatedly in both experimental and predicted datasets supports the idea that *SARM1* could be a key player in sALS, a notion also backed by several recent studies (Xie et al., 2014; Fogh et al., 2014; Consortium, 2025).

On top of *SARM1*, we noticed that four other genes—*OPHN1*, *BPTF*, *BMI1P1*, and *AR*—appeared repeatedly, carrying both significant and frequent SNPs according to the model. Since these genes have also been mentioned in previous studies as associated with sALS (Proaño et al., 2022; Zapata et al., 2022), it seems reasonable to think they play important roles in how the disease progresses and might be worth following up in future experiments.

When looking at the chromosomes, some patterns are clear from Figure 8. Chromosome 18, for example, has a lot of predicted sALS SNPs. The way they cluster does not seem random and might suggest that some regions have a bigger role in disease risk. These findings make it more likely that the predicted SNPs are biologically meaningful and show that machine learning can highlight parts of the genome that traditional analyses might have missed.

These observations suggest that the predicted SNPs and their genes are more than just numbers. They seem to reflect real biological processes that match what is already known about sALS, which adds confidence in the predictions and shows the potential of combining machine learning with traditional genetic data to learn more about rare, complex diseases like sALS.

Beyond its performance, the conceptual architecture of our protocol represents a methodological advance for the study of rare diseases. Its generic nature—validated across multiple pathologies and independent of extensive resources—makes it a reproducible framework adaptable to other contexts with limited data, thereby filling a methodological gap in the bioinformatics toolkit.

## 6 Conclusion

This study demonstrates not only the utility of machine learning for biomarker discovery in sALS but also, and more importantly, proposes a novel methodological framework specifically designed for rare diseases. The reformulation of the task as a similarity problem, combined with biologically informed feature engineering, provides a scalable solution to the challenge of limited genetic data.

It provides evidence that machine learning can uncover genetic SNPs associated with sALS. By assuming that nearby SNPs may have similar effects, our Random Forest model highlighted 1,890 candidate SNPs, some statistically significant and others repeatedly predicted. Genes like *SARM1*, *OPHN1*, *AR*, and *BPTF* were particularly noteworthy, and chromosome 18 appeared to be of special interest. These results offer new insights into sALS genetics and may help guide future work on early diagnosis and potential therapeutic targets. Even though this analysis focused on sALS, the approach could be applied to other rare diseases with limited genetic data. Identifying SNPs that may have biological relevance could suggest important pathways, indicate early diagnostic biomarkers, and highlight targets for experimental follow-up. Although confirmation through *in vitro* experiments is still needed, this approach offers a solid starting point for exploring the genetics of rare diseases.

## Data Availability

The original contributions presented in the study are publicly available. The SNP dataset and associated Python scripts have been deposited in Zenodo at: https://doi.org/10.5281/zenodo.18789012.
